# Genome-Wide Identification, Cloning and Functional Analysis of the Zinc/Iron-Regulated Transporter-Like Protein (*ZIP*) Gene Family in Trifoliate Orange (*Poncirus trifoliata* L. Raf.)

**DOI:** 10.3389/fpls.2017.00588

**Published:** 2017-04-19

**Authors:** Xing-Zheng Fu, Xue Zhou, Fei Xing, Li-Li Ling, Chang-Pin Chun, Li Cao, Mark G. M. Aarts, Liang-Zhi Peng

**Affiliations:** ^1^Citrus Research Institute, Southwest UniversityChongqing, China; ^2^Citrus Research Institute, Chinese Academy of Agricultural SciencesChongqing, China; ^3^Laboratory of Genetics, Wageningen UniversityWageningen, Netherlands

**Keywords:** citrus, zinc deficiency, iron deficiency, *ZIP* gene, yeast complementation

## Abstract

Zinc (Zn) and iron (Fe) deficiency are widespread among citrus plants, but the molecular mechanisms regarding uptake and transport of these two essential metal ions in citrus are still unclear. In the present study, 12 members of the Zn/Fe-regulated transporter (ZRT/IRT)-related protein (*ZIP*) gene family were identified and isolated from a widely used citrus rootstock, trifoliate orange (*Poncirus trifoliata* L. Raf.), and the genes were correspondingly named as *PtZIPs* according to the sequence and functional similarity to *Arabidopsis thaliana ZIPs*. The 12 *PtZIP* genes were predicted to encode proteins of 334–419 amino acids, harboring 6–9 putative transmembrane (TM) domains. All of the PtZIP proteins contained the highly conserved ZIP signature sequences in TM-IV, and nine of them showed a variable region rich in histidine residues between TM-III and TM-IV. Phylogenetic analysis subdivided the PtZIPs into four groups, similar as found for the *ZIP* family of *A. thaliana*, with clustered PtZIPs sharing a similar gene structure. Expression analysis showed that the *PtZIP* genes were very differently induced in roots and leaves under conditions of Zn, Fe and Mn deficiency. Yeast complementation tests indicated that *PtIRT1, PtZIP1, PtZIP2, PtZIP3*, and *PtZIP12* were able to complement the *zrt1zrt2* mutant, which was deficient in Zn uptake; *PtIRT1* and *PtZIP7* were able to complement the *fet3fet4* mutant, which was deficient in Fe uptake, and *PtIRT1* was able to complement the *smf1* mutant, which was deficient in Mn uptake, suggesting their respective functions in Zn, Fe, and Mn transport. The present study broadens our understanding of metal ion uptake and transport and functional divergence of the various *PtZIP* genes in citrus plants.

## Introduction

Zinc (Zn) and iron (Fe) are essential micronutrients for plant growth and development. As a cofactor for many enzymes and transcription factors, Zn has been involved in a wide range of cellular processes such as photosynthesis, nucleic acid and lipid metabolism, protein synthesis, and membrane stability (Broadley et al., 2007; Sinclair and Krämer, 2012). Fe functions as a catalyst for many cellular reactions such as the electron transport of photosynthesis and respiration, and chlorophyll biosynthesis (Jeong and Guerinot, 2009). Shortage or excess of Zn and Fe would cause severe nutritional disorders. To cope with this issue, plants have developed a tightly regulated cellular homeostasis system to balance the uptake, distribution and utilization of these metal ions (Clemens, 2001; Grotz and Guerinot, 2006). In this system, various members of the zinc/iron-regulated transporter (ZRT/IRT)-related protein (ZIP) family, natural resistance associated macrophage protein (NRAMP) family, cation diffusion facilitator (CDF) family, yellow stripe-like (YSL) family, major facilitator super family (MFS), P_1B_-type heavy metal ATPase (HMA) family, vacuolar iron transporter (VIT) family, and the cation exchange (CAX) family have been shown to play key roles (Vigani et al., 2013; Boutigny et al., 2014; Bashir et al., 2016).

The ZIP family members are thought to be important transporters for uptake and transport of Zn, Fe, Mn, and Cu (Eide et al., 1996; Zhao and Eide, 1996a,b; Guerinot, 2000; Grotz and Guerinot, 2006). The first ZIP protein, AtIRT1, has been identified in *Arabidopsis thaliana*, by functional expression in yeast (Eide et al., 1996). Subsequently, other plant ZIP family members were reported in tomato (Eckhardt et al., 2001), soybean (Moreau et al., 2002), *A. thaliana* (Milner et al., 2013), rice (Ishimaru, 2005; Yang et al., 2009; Lee et al., 2010b), *Medicago truncatula* (López-Millan et al., 2004), barley (Pedas et al., 2009) and maize (Li et al., 2013). In general, the ZIP proteins are consist of 309–476 amino acid residues with eight potential transmembrane (TM) domains and a similar membrane topology in which the N- and C-terminal ends of the protein are located on the outside surface of the plasma membrane. Between TM-III and TM-IV there is a variable region, which contains a potential metal-binding domain and is rich in histidine residues (Guerinot, 2000). Functionality of some *AtZIP* genes in mineral uptake has been demonstrated. Knockout of *AtIRT1* in *A. thaliana* resulted in Fe deficiency, accompanied by cell differentiation defects (Henriques et al., 2002; Vert et al., 2002), while *AtIRT2* expression in yeast restored the growth of Fe and Zn transport yeast mutants and enhanced Fe uptake (Vert et al., 2001). Overexpression of *AtIRT3* increased the accumulation of Zn in the shoots and Fe in the roots of transgenic plants (Lin et al., 2009), while knock-out mutants of *AtZIP1* and *AtZIP2* exhibited defects in remobilization and translocation of Mn and Zn (Milner et al., 2013). Although the gramineous plants, unlike nongraminaceous plants, mainly depend on a mineral-chelation strategy (Strategy II) to acquire Fe (Kobayashi and Nishizawa, 2012), the *ZIP* genes of rice and maize, such as *OsZIP4, OsZIP5, OsZIP8, ZmIRT1*, and *ZmZIP3*, have been shown to be involved in Fe or Zn transport and distribution (Ishimaru et al., 2007; Lee et al., 2010a,b; Li et al., 2015). All these studies suggest that the expression of the most *ZIPs* was induced by Zn, Fe, or Mn deficiency, and that regulating the endogenous expression of *ZIPs* is essential to maintain cellular metal homeostasis, since these genes are involved in root metal uptake as well as metal transport and distribution among plant organs.

Because of the importance of ZIPs in metal ion uptake, transport and distribution, much research in recent years has focused on cloning and characterizing their functions in crop plants. However, there is still little information regarding *ZIPs* in perennial plants, such as citrus. Zn and Fe deficiencies are common in citrus trees, resulting in severe chlorosis of leaves, impaired tree vigor, and reduction of fruit set, yield, size, and quality (Fu et al., 2016). As a major rootstock, trifoliate orange (*Poncirus trifoliata* L. Raf.) is directly responsible for nutrient uptake from soil; cloning and functionally analyzing *ZIPs* of this rootstock could facilitate significantly in the understanding of the underlying Zn and Fe uptake mechanisms. Moreover, sequencing and assembly of a citrus genome (http://citrus.hzau.edu.cn/orange/; Xu et al., 2013) provided the opportunity to identify and isolate such genes at the genome level. In the present study, we cloned 12 ZIP members of trifoliate orange and conducted detailed sequence analysis including multiple sequence alignment, phylogenetic tree construction, chromosomal location, and prediction of the transmembrane domains, gene structure, and subcellular localization. We have also characterized their expression patterns in roots and leaves under Zn, Fe, and Mn deficiency, as well as the metal selectivity and uptake activity by functional complementation of yeast mutants. The results provide us with systematic information regarding the possible functions of each of these *PtZIP* genes and lay the foundation for further studies.

## Materials and methods

### Plant materials and treatments

The outer and inner seed coats of trifoliate orange (*Poncirus trifoliata* L. Raf.) seeds were removed and seeds were germinated at a temperature of 28°C and a relative humidity of 70% under darkness for 7 d. Thereafter, the germinated seedlings were transferred to a hydroponic solution composed of 2 mM Ca(NO_3_)_2_, 3 mM KNO_3_, 0.5 mM NH_4_H_2_PO_4_, 1 mM MgSO_4_, 20 μM H_3_BO_3_, 10 μM MnSO_4_, 5 μM ZnSO_4_, 1 μM CuSO_4_, 1 μM H_2_MoO_4_, and 50 μM Fe-EDTA at 25°C and 16 h photoperiod (50 μmol m^−2^ s^−1^) for 30 d of normal growth. For Zn-, Fe-, and Mn-deficient (-Zn, -Fe, and -Mn) treatments, the trifoliate orange seedlings were transferred to new hydroponic solutions without ZnSO_4_, Fe-EDTA, or MnSO_4_, respectively, and those grown in normal medium were used as a control (CK). After 7, 12, and 20 d of nutrient-deficient treatments, the roots and leaves were sampled and immediately frozen in liquid nitrogen, then stored at −80°C until use.

### Identification and cloning of *ZIP* genes in trifoliate orange

*ZIP* genes of sweet orange (*Citrus sinensis*) were first identified by a BLASTP search of the sweet orange genome database (http://citrus.hzau.edu.cn/orange/; Xu et al., 2013) using 15 of the known *A. thaliana* ZIP proteins (AT3G12750.1, AT5G59520.1, AT2G32270.1, AT1G10970.1, AT1G05300.1, AT2G30080.1, AT2G04032.1, AT5G45105.2, AT4G33020.1, AT1G31260.1, AT1G55910.1, AT5G62160.1, AT4G19690.2, AT4G19680.2, and AT1G60960.1) and 12 of the known rice (*Oryza sativa* L.) ZIP proteins (AY302058.1, AY302059.1, AY323915.1, AB126089.1, AB126087.1, AB126088.1, AB126090.1, AY275180.1, AY324148.1, AY281300.1, AB070226.1, and AB126086.1) as query sequences. The results were filtered at the score value of ≥100 and an *e* ≤ *e*^−10^ (Kumar et al., 2011). After removing duplicate and overlapping genes, the remaining non-overlapping genes were further analyzed for their potential transmembrane domains using TMHMM (Krogh et al., 2001).

To clone all *ZIP* genes from trifoliate orange, primers were designed to amplify the full open-reading frame (ORF) according to identified *ZIP* sequences of sweet orange. Two Gateway recombination sites, attB1- GGGGACAAGTTTGTACAAAAAAGCAGGCTTC and attB2- GGGGACCACTTTGTACAAGAAAGCTGGGTC, were incorporated into the 5′-end of forward and reverse primers, respectively, as described in the Gateway technology instructions (Invitrogen). The cDNAs derived from Zn- or Fe-deficient leaves and roots of trifoliate orange served as templates (see below). The amplified *att*B-PCR fragments were then cloned into the entry vector pDONR221 (Invitrogen) to generate entry clones by BP recombination reaction with the Gateway BP Clonase II enzyme (Invitrogen). The resulting pDONR221-*ZIP*s entry clones were transformed into DH5α *E. coli* competent cells and then sequenced at the Beijing Genomics Institute (BGI, China). The *ZIP* genes isolated from trifoliate orange were designated *PtZIP* genes.

### Sequence analysis of *PtZIP* genes

The putative ORFs of *PtZIP* genes were analyzed using NCBI ORF finder (https://www.ncbi.nlm.nih.gov/orffinder/). The translated PtZIP protein sequences were submitted to ExPASy (http://web.expasy.org/compute_pi/) to calculate molecular weights (MWs) and isoelectric points (pIs). Gene structure analysis was performed by using the Gene Structure Display Server (GSDS, http://gsds.cbi.pku.edu.cn/) program (Guo et al., 2007). The chromosomal positions of the *PtZIP* genes were provided by the sweet orange genome database, and the MapInspect software (http://mapinspect.software.informer.com) was used to draw the location images. Potential transmembrane domains in each PtZIP protein were identified using TMHMM (Krogh et al., 2001). The signal peptides of PtZIPs were identified with SignalP 4.1 (http://www.cbs.dtu.dk/services/SignalP/; Petersen et al., 2011). The subcellular localizations of the PtZIP proteins were predicted using the subCELlular LOcalization predictor (CELLO v.2.5; http://cello.life.nctu.edu.tw/; Yu et al., 2006) and ProtComp v. 9.0 online (http://www.softberry.com/berry.phtml?topic=protcomppl&group=programs&subgroup=proloc).

Multiple sequence alignment of the PtZIP and AtZIP proteins was performed with the ClustalW (Thompson et al., 1994) integrated in MEGA version 6 (Tamura et al., 2013). The generated files were then evaluated using MEGA 6 software to construct a phylogenetic tree based on the neighbor-joining method with 1,000 replicates of bootstrap analysis, with the other parameters set as described by Tamura et al. (2013).

### Quantitative real-time RT-PCR (qPCR) analysis

Total RNA was isolated from the leaves and roots of control (CK), -Fe, -Zn, and -Mn treated trifoliate orange seedlings using the RNAprep pure plant kit (Tiangen Biotech Co., Ltd., Beijing, China). Then 1 μg of the total RNA was used for cDNA synthesis with an iScript™ cDNA synthesis kit (Bio-Rad) according to the manufacturer's instructions. Specific primers of *PtZIP* genes were designed using the online primer-blast program in the NCBI website, while *Actin* (Cs1g05000.1) was used as a reference gene to normalize the relative expression levels of the target genes. qPCR was performed by using a Bio-Rad CFX Connect Real-Time system. Each reaction contained 5 μL iTaq Universal SYBR Green Supermix dye (Bio-Rad), 1 μL cDNA, and 0.2 μM gene-specific primers in a final volume of 10 μL. The PCR condition was set up as follow: 95°C for 30 s, followed by 40 cycles of 95°C for 5 s and 55°C–60°C for 30 s. Two biological replicates and three technical replicates were performed for each treatment.

### Yeast complementation

The pDONR221-*PtZIP* entry clones were recombined into the yeast expression vector pFL613 (Dräger et al., 2004) by an LR recombination reaction with Gateway LR Clonase II enzyme (Invitrogen). The resulting pFL613-*PtZIP* constructs were then transformed into three yeast strains, the Zn uptake-defective mutant *zrt1zrt2* ZHY3 (*MAT*α *ade6 can1 his3 leu2 trp1 ura3 zrt1::LEU2 zrt2::HIS3*; Zhao and Eide, 1996b), the Fe uptake-defective mutant *fet3fet4* DEY1453 (*MATa/MAT*α *ade2/*+ *can1/can1 his3/his3 leu2/leu2 trp1/trp1 ura3/ura3 fet3-2::HIS3/fet3-2::HIS3 fet4-1::LEU2/fet4-1::LEU2*; Eide et al., 1996), and the Mn uptake-defective mutant *smf1* (*MAT*α *his3 ade2 leu2 trp1 ura3 smf1::*URA3*ura3::TRP1*; Thomine et al., 2000). The empty vector pFL613 was used as a negative control, and *AtZIP4, AtIRT1*, and *AtZIP7* were used as positive controls for complementing *zrt1zrt2, fet3fet4*, and *smf1*, respectively (Eide et al., 1996; Assunção et al., 2010; Milner et al., 2013). In addition, the wild type strain DY1455 harboring pFL613 was used as another positive control. The complementation tests were performed using a slight modification of the method described by Milner et al. (2013). Briefly, transformed cells were selected and cultured on synthetic complete and dropout mix (without uracil) medium (SC-URA) plus 0.1 mM ZnSO_4_ for *zrt1zrt2* or 0.1 mM Fe_2_(SO_4_)_3_ for *fet3fet4*. Then 5-μL aliquots of each yeast culture at optical densities (OD_600_) of 1.0, 0.1, 0.01, and 0.001 were spotted onto the specific Zn-, Fe-, and Mn-limiting media for *zrt1zrt2, fet3fet4*, and *smf1* mutants, respectively. The Zn-limiting medium contained SC-URA plus 1.0 mM ethylenediaminetetraacetic acid (EDTA, Sigma), 0.4 or 0.6 mM ZnSO_4_, and 10 mM MES (pH 5.0). The Fe-limiting medium contained SC-URA plus 0.01 or 0.02 mM bathophenanthroline disulphonic acid (BPDS, Sigma), and 10 mM MES (pH 6.0). The Mn-limiting medium contained SC-URA plus 10 or 20 mM ethylene glycol-*bis*-β-aminoethylether-*N,N,N*′,*N*′-tetreacetic acid (EGTA, Sigma) and 50 mM MES (pH 6.0). Yeast complementation was also tested by measuring the OD_600_ in the described Zn- (1.0 mM EDTA and 0.4 mM ZnSO_4_), Fe- (0.01 mM BPDS) and Mn- (10 mM EGTA) limiting liquid media. To conduct this experiment, 15 mL liquid medium was initially inoculated with a 100-μL single-colony preculture at OD_600_ = 1, then shaken at 200 rpm and 30°C. After 20, 32, 44, and 56 h growth, 2 mL of the 15 mL culture was utilized to measure the OD_600_. Three independent repeats were performed.

## Results

### Isolation of *PtZIP* genes

To genome-wide identify all *ZIP* genes in citrus, a BLASTP search of the sweet orange genome database was performed by using known *A. thaliana* and rice ZIP proteins as queries. As a result, after removal of the overlapping genes and alternative splice forms of the same gene, 13 sweet orange genes were identified, which are most similar to the ZIP sequences used as query. Further bioinformatics analysis shows that 12 of them contained transmembrane domains (TMs) and would be localized to the plasma membrane as predicted. This is consistent with known characteristics of *ZIP* genes. Therefore, these 12 genes were considered to be true citrus *ZIP* genes. Subsequently, the *ZIP* genes were PCR-amplified, cloned, and sequenced from trifoliate orange using sweet orange sequences as references, and were correspondingly named as *PtZIPs* according to the sequence and functional similarity to *A. thaliana ZIPs* (Table 1). The length of the *PtZIPs* ORF ranged from 1,005 bp (*PtZIP2*) to 1,260 bp (*PtZIP9*) with 2–4 exons, encoding 334–419 amino acids and harboring 6–9 putative TMs. The predicted MV and pI of PtZIP proteins ranged from 36.5 to 45.1 kDa and 5.5 to 8.0, respectively (Table 1).

**Table 1:** ***PtZIP* genes encoding ZIP proteins along with their molecular details**.

**Gene**	**Gene ID^a^**	**ORF**	**No. exon**	**Protein length**	**Mw (kDa)**	**pI**	**Chromosome**	**TM domains**	**Predicted subcellular localization**
*PtIRT1*	Cs2g10720.1	1062	3	353	37.8	7.4	2	9	Plasma membrane
*PtZIP1*	orange1.1t03275.1	1056	3	351	37.5	5.8	Unknown	6	Plasma membrane
*PtZIP2*	Cs8g18810.1	1005	2	334	36.5	5.7	8	9	Plasma membrane
*PtZIP3*	Cs6g11470.1	1068	3	355	38.0	6.1	6	9	Plasma membrane
*PtZIP5*	orange1.1t03274.1	1080	3	359	38.5	6.2	Unknown	8	Plasma membrane
*PtZIP6*	Cs8g20480.1	1053	2	350	37.7	6.8	8	8	Plasma membrane
*PtZIP7*	Cs7g12260.1	1044	3	347	37.5	7.3	7	8	Plasma membrane
*PtZIP9*	Cs4g18450.1	1260	4	419	45.1	6.4	4	6	Plasma membrane
*PtZIP11*	Cs4g08930.1	1044	3	347	37.1	5.5	4	9	Plasma membrane
*PtZIP12*	Cs2g11610.1	1074	3	357	38.3	6.3	2	7	Plasma membrane
*PtZIP13*	Cs2g11620.1	1086	3	361	38.8	8.0	2	7	Plasma membrane
*PtZIP14*	Cs6g11460.1	1077	3	358	38.4	6.2	6	9	Plasma membrane

*MW, molecular weight, kDa; pI, isoelectric point; TM domains, number of transmembrane domains*.

a *The Gene ID from the Sweet Orange genome database (http://citrus.hzau.edu.cn/orange/)*.

### Sequence analysis of *PtZIP* genes

**Figure 1:**
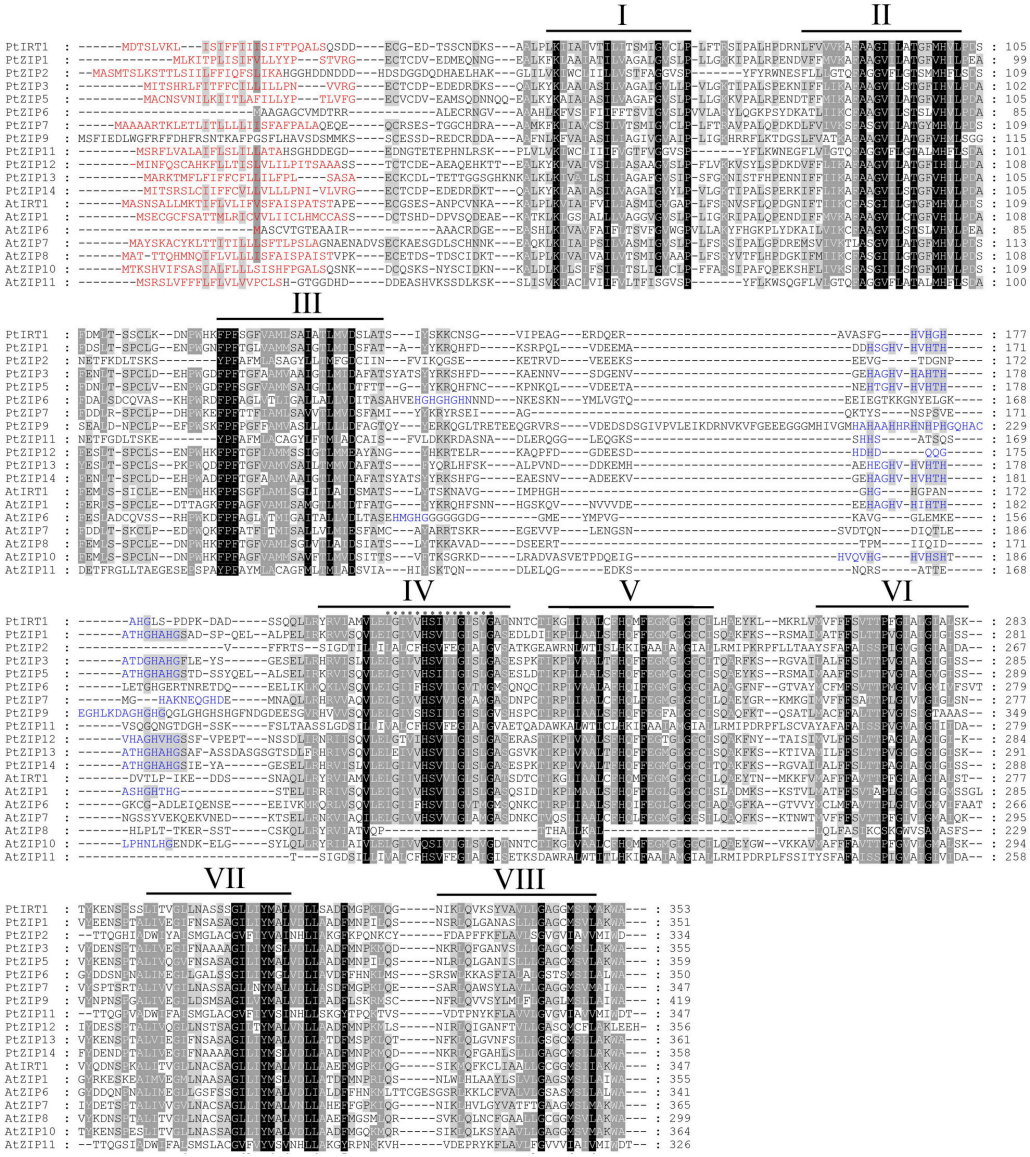
**Multiple sequence alignment of PtZIP and AtZIP proteins**. Twelve PtZIP proteins isolated from trifoliate orange and seven representative AtZIP proteins from *Arabidopsis* were aligned using ClustalW. Similar amino acids are indicated by dark or light shading, while signal peptides in the N-terminal end are highlighted in red. Transmembrane (TM) domains were shown as lines above the sequences and numbered I to VIII. The variable region rich in histidine residues between TM-III and TM-IV is highlighted in blue. The 15 ZIP signature sequences in TM-IV domain are marked with asterisks.

**Figure 2:**
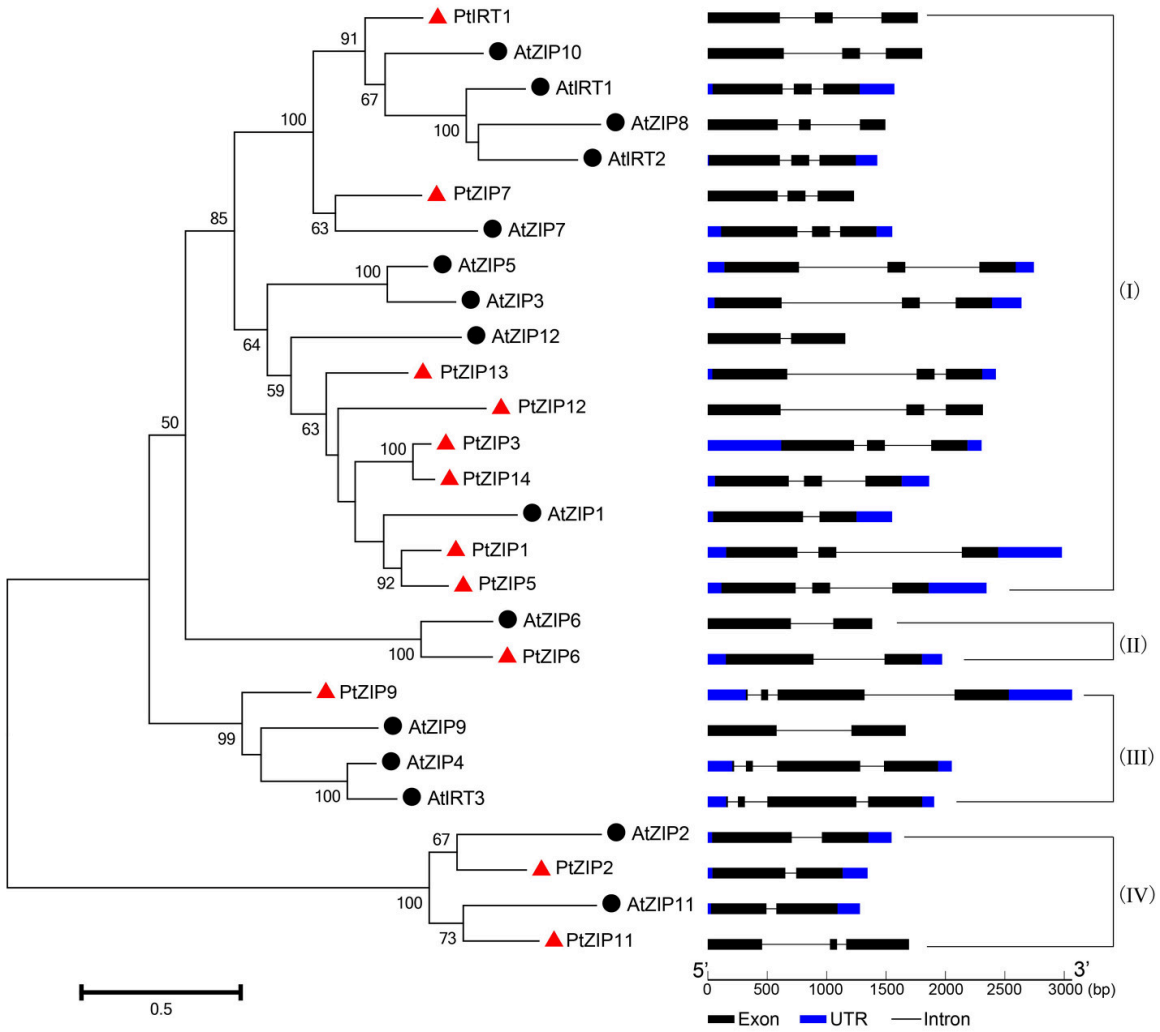
**Phylogenetic tree and gene structure of PtZIP and AtZIP proteins**. Twelve of the PtZIP proteins (marked with red triangles) and 15 of the AtZIP proteins (marked with black spots) were used to construct the neighbor-joining tree. Bootstrap values above 50 and supporting a node used to define a cluster are indicated. The structures of each gene were analyzed online using the Gene Structure Display Server and displayed according to the order of the phylogenetic relationship. The black boxes, blue boxes and lines represent exons, UTRs and introns, respectively, and their lengths are shown proportionally. The proteins were divided into four groups (I to IV) according to their phylogenetic relationships.

**Figure 3:**
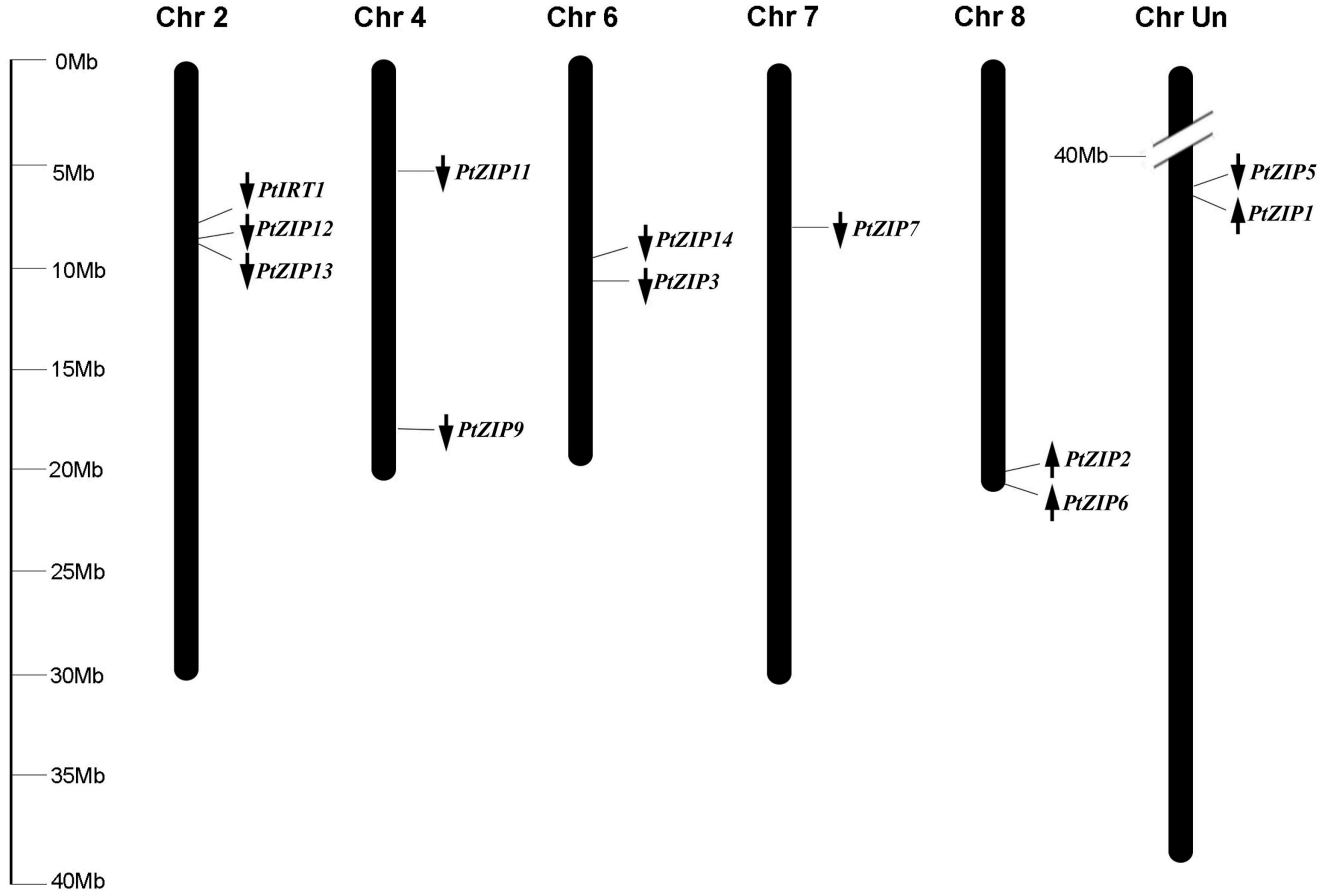
**Distribution of *PtZIP* genes on the chromosomes**. The chromosomal positions of the *PtZIP* genes were mapped according to the information available in the sweet orange genome database. The arrows indicate transcription direction. The chromosome numbers are indicated at the top of each chromosome. The scale is in megabases (Mb).

Multiple sequence alignment showed high similarity of PtZIP and AtZIP proteins, as well as a region variable in length containing a metal-binding domain rich in histidine residues between TM-III and TM-IV in all PtZIPs, except for PtZIP2 (Figure 1). Moreover, all 12 PtZIPs were predicted to be plasma membrane localized by CELLO and ProtComp predictor (Table 1), and 10 of them (excluding PtZIP6 and PtZIP9) contained a visible signal peptide for the secretory pathway at their N-terminal end (Figure 1). Phylogenetic analysis revealed that the PtZIPs were divided into four groups, the same as for AtZIPs. For nearly all AtZIPs, putative orthologues were found in trifoliate orange, except for a few closely related AtZIPs, which corresponded to only one PtZIP. The cluster with AtIRT1, AtIRT2, AtZIP8, and AtZIP10 only contained one trifoliate orange orthologue, PtIRT1. And the cluster with AtZIP4, AtZIP9, and AtIRT3, only contained PtZIP9. For the cluster containing AtZIP1, AtZIP3, AtZIP5, and AtZIP12, the closest orthologues could not be assigned, as no less than six PtZIPs resembled these four AtZIPs (Figure 2). The ZIP proteins in the same cluster often shared a similar gene structure (Figure 2). The 12 *PtZIP* genes could be assigned to six of the nine sweet orange chromosomes (Figure 3). The chromosomal location of two *PtZIP* genes could not be determined. It seems that some *PtZIP* genes physically cluster to very close regions, for example, *PtIRt1, PtZIP12*, and *PtZIP13* on chromosome 2, *PtZIP3* and *PtZIP14* on chromosome 6, *PtZIP2* and *PtZIP6* on chromosome 8, and *PtZIP1* and *PtZIP5* on an unassigned chromosome. Of these, *PtZIP12* and *PtZIP13, PtZIP3* and *PtZIP14*, and *PtZIP1* and *PtZIP5* are neighboring on genome and also grouped on phylogenetic tree. It is suggested that these genes might be tandem duplicated in the evolutionary history of trifoliate orange.

### Expression patterns of *PtZIP* genes

**Figure 4:**
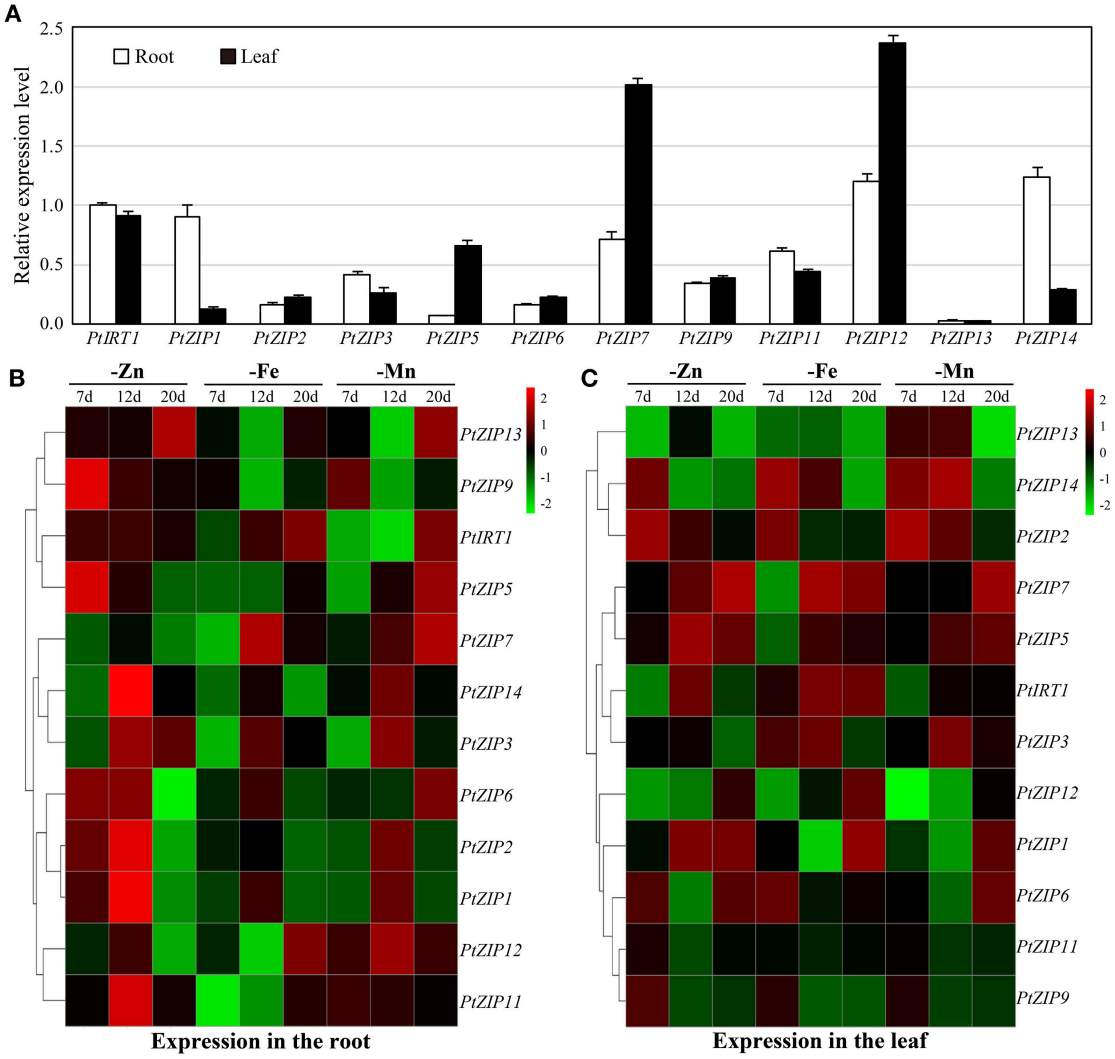
**Expression patterns of *PtZIP* genes in roots and leaves under normal conditions or Zn, Fe and Mn deficiency. (A)** qPCR analysis of *PtZIP* gene expression in roots and leaves of 40 d-old trifoliate orange seedlings grown on normal nutrient solution. Data are the means ± SE of three technical replicates. **(B)** Heat map showing the expression patterns of *PtZIP* genes following treatment to induce Zn, Fe and Mn deficiency for 7, 12, and 20 d. Relative expression levels of *PtZIP* genes in roots and leaves were analyzed by qPCR and normalized to *Actin* (Cs1g05000.1). The relative expression values in samples those grown under normal conditions for 7, 12, and 20 d were used as controls. The fold change was then calculated as (*PtZIP* expression under treatment)/(*PtZIP* expression under control). The cluster 3.0 software was then used to generate heat maps based on the ${\text{log}}_{2}^{\text{fold }change}$ data. Green and red indicates lower and higher transcriptional levels of *PtZIP* genes, respectively.

To better understand the functions of these *PtZIP* genes, their expression patterns were investigated under Zn-, Fe-, and Mn-sufficient and -deficient conditions in different organs (root and leaf) of trifoliate orange. Under sufficient conditions, *PtIRT1, PtZIP1, PtZIP5, PtZIP7, PtZIP12*, and *PtZIP14* were higher expressed than the others in either roots or shoots. Expression levels of *PtZIP1, PtZIP3, PtZIP11*, and *PtZIP14* were relatively high in roots, while *PtZIP5, PtZIP7*, and *PtZIP12* were higher in leaves (Figure 4A). Under deficient conditions, expression of each *PtZIP* gene was determined at three time points (7, 12, and 20 d after treatment), and those highly expressed (${\text{log}}_{2}^{\text{fold change}}$ > 1) at least two times were considered as differentially induced by the treatment. As shown in Figures 4B,C, the expression of *PtIRT1, PtZIP1, PtZIP2, PtZIP3, PtZIP5, PtZIP6*, and *PtZIP9* was significantly induced in Zn-deficient roots, while *PtZIP1, PtZIP2, PtZIP5, PtZIP6*, and *PtZIP7* were significantly induced in Zn-deficient leaves. Additionally, the expression of *PtIRT1* and *PtZIP7* was significantly induced in Fe-deficient roots, while that of *PtIRT1, PtZIP3, PtZIP5, PtZIP7*, and *PtZIP14* was significantly induced in Fe-deficient leaves. Furthermore, the expression of *PtZIP7, PtZIP11*, and *PtZIP12* was significantly induced in Mn-deficient roots, while that of *PtZIP2, PtZIP5, PtZIP13*, and *PtZIP14* was significantly induced in Mn-deficient leaves. Taken together, the *PtZIP* members exhibited very different expression profiles in different organs and in response to different metal-deficiency treatments.

### Complementation of *PtZIP* genes in yeast mutants

**Figure 5:**
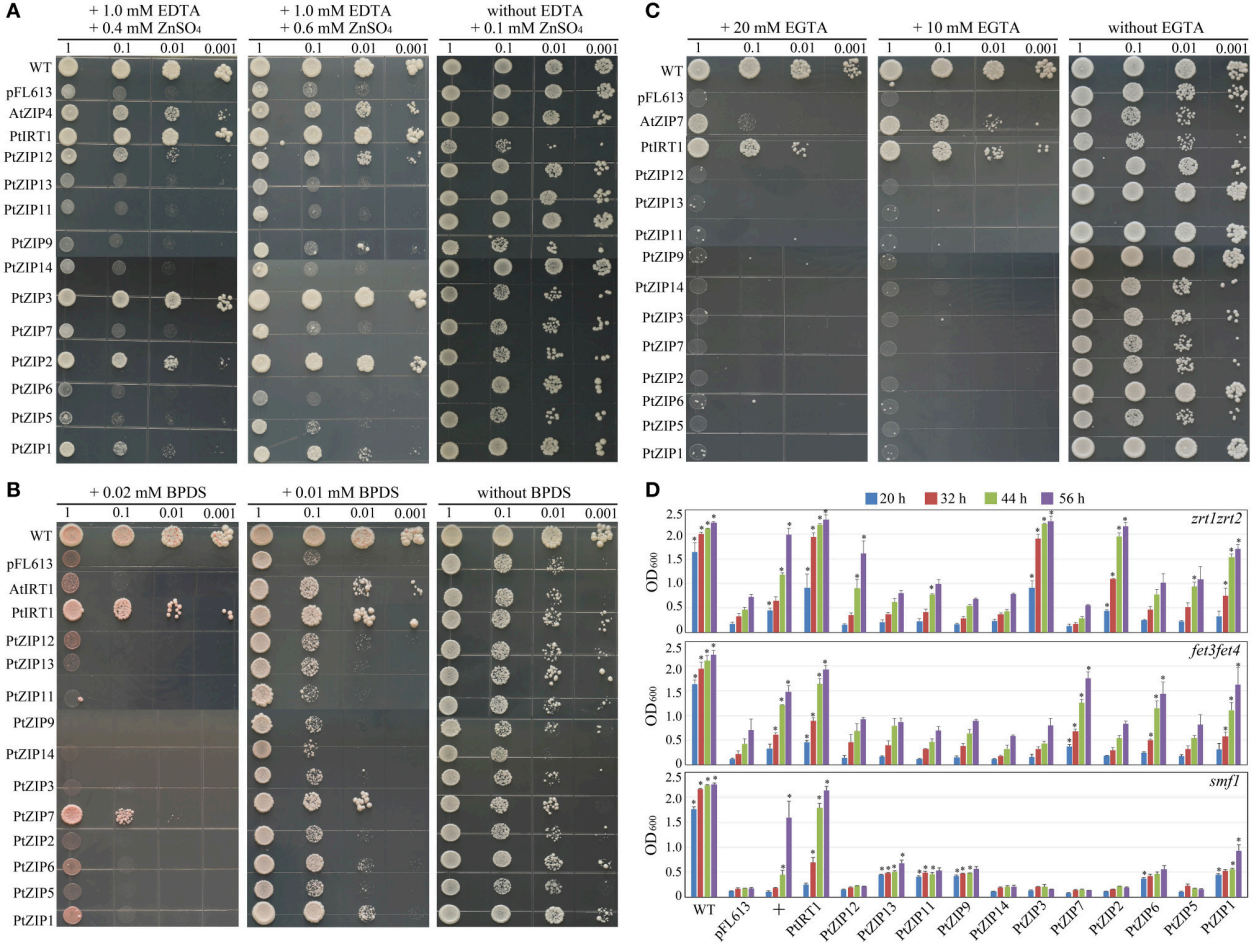
**Complementation of yeast metal uptake-defective mutants with *PtZIP* genes on selective medium**. Yeast mutants defective in the uptake of Zn (*zrt1zrt2*), Fe (*fet3fet4*), and Mn (*smf1*) were transformed with any one of the 12 *PtZIPs*, the empty vector pFL613 (negative control), or the *AtZIP4* (positive control for *zrt1zrt2*), *AtIRT1* (positive control for *fet3fet4*) and *AtZIP7* (positive control for *smf1*). To test the complementary ability of the genes, 5 μL of transformed yeast cells with OD_600_ values of 1.0, 0.1, 0.01, and 0.001 were spotted on plates containing different metal limiting medium and grown for 3–5 d at 30°C. **(A)** Transformed *zrt1zrt2* cells on synthetic complete and dropout mix (without Uracil) medium (SC-URA) plus 0.1 mM ZnSO_4_ (control) or SC-URA plus 1.0 mM EDTA and 0.4/0.6 mM ZnSO_4_ (Zn limited with EDTA). **(B)** Transformed *fet3fet4* cells on SC-URA without BPDS (control) or SC-URA plus 0.01/0.02 mM BPDS (Fe limited with BPDS). **(C)** Transformed *smf1* cells on SC-URA without EGTA (control) or SC-URA plus 10/20 mM EGTA (Mn limited with EGTA). **(D)** Transformed yeast cells (100 μL) with an OD_600_ of 1.0 were initially inoculated into 15 mL liquid SC-URA medium supplemented with 1.0 mM EDTA and 0.4 mM ZnSO_4_ (for testing *zrt1zrt2*), 0.01 mM BPDS (for testing *fet3fet4*), and 10 mM EGTA (for testing *smf1*). After 20, 32, 44, and 56 h of growth at 200 rpm and 30°C, the OD_600_ of 2 mL from the 15 mL culture was measured. Data are the means ± SE of three independent repeats. + under the horizontal axis indicates the positive controls, AtZIP4, AtIRT1 and AtZIP7 for zrt1zrt2, fet3fet4, and smf1, respectively. ^*^ indicates the values are significantly different from those associated with pFL613 at *P* < 0.05 by LSD testing.

To determine the metal transport specificities of the PtZIPs, each *PtZIP* gene was expressed in Zn uptake-defective (*zrt1zrt2*), Fe uptake-defective (*fet3fet4*), and Mn uptake-defective (*smf1*) yeast mutants. As shown in Figure 5A, the *zrt1zrt2* mutant transformed with *PtZIP* genes or the pFL613 empty vector (negative control) grew well on Zn-sufficient control medium (plus 0.1 mM ZnSO_4_), but on Zn-limited medium (plus 1.0 mM EDTA and 0.6/0.4 mM ZnSO_4_) only those transformed with *AtZIP4* (positive control), *PtIRT1, PtZIP1, PtZIP2, PtZIP3*, and *PtZIP12* showed normal growth relative to the negative control and other transformants, suggesting that these five *PtZIPs* are able to complement *zrt1zrt2* mutant and transport Zn. Similarly, in the presence of 0.01 mM BPDS, the *fet3fet4* mutant could be complemented by *AtIRT1* (positive control), *PtIRT1, PtZIP1*, and *PtZIP7* and to a lesser extent by *PtZIP6* (Figure 5B). When Fe availability was more strictly limited with 0.02 mM BPDS, the *PtIRT1* and *PtZIP7* genes still complemented the *fet3fet4* mutant (Figure 5B). Only *PtIRT1* and the positive control (*AtZIP7*) were able to complement the *smf1* mutant on Mn-limited medium (plus 10 or 20 mM EGTA; Figure 5C). To provide more evidence, the complementation experiment was also performed on liquid cultures. The OD measurements perfectly supported the drop spotting assay results as shown in Figure 5D. The *zrt1zrt2* mutant transformed with *PtIRT1, PtZIP1, PtZIP2, PtZIP3*, and *PtZIP12* had significantly higher ODs after 20, 32, 44, and 56 h of growth than the pFL613 empty vector control, which almost reach the same values as the WT at the last time point (56 h). Similarly, the ODs of *fet3fet4* transformed with *PtIRT1, PtZIP1, PtZIP6*, and *PtZIP7* and the OD of *smf1* transformed with *PtIRT1* were significantly higher than the OD of the negative control at all four time points. Although the OD of *smf1* transformed with *PtZIP1, PtZIP9, PtZIP11*, and *PtZIP13* were also significantly higher than the control, their OD did not increase as much as expected for full complementation after 20 h of growth.

## Discussion

Although *ZIP* genes have been reported in several plant species, to the best of our knowledge, this is the first study to systematically identify, clone and characterize 12 *PtZIPs* from trifoliate orange. The numbers of *PtZIPs* in trifoliate orange or citrus is the same as that in rice, but less than in *A. thaliana*. Sequence analysis shows that the PtZIP proteins contain several, if not all, characteristics of the known ZIP family members (Eng et al., 1998; Guerinot, 2000). Specifically, the 12 identified *PtZIP* genes encode polypeptides of 334–419 amino acids, all within the range of known plant ZIPs (Guerinot, 2000). All PtZIPs were predicted to be localized to the plasma membrane (Table 1) as it is known for AtIRT1, OsIRT1, HvIRT1, OsZIP4, and OsZIP5 (Bughio et al., 2002; Vert et al., 2002; Ishimaru, 2005; Pedas et al., 2008; Lee et al., 2010b). It confirms their putative role in metal ion uptake or transport. In addition, 6–9 putative TMs were identified in the PtZIP proteins, using TMHMM (Table 1). Although not all have 8 TMs as proposed by Guerinot (2000), this is closely consistent with the number of TMs found in ZmZIPs of maize (Li et al., 2013). A variable region between TM-III and TM-IV was also found in almost all PtZIPs except PtZIP2 (Figure 1). This region is predicted to be directed toward the cytoplasmic side of the plasma membrane and it is rich in histidine residues, thus providing a cytoplasmic metal ion binding site (Eng et al., 1998; Guerinot, 2000). PtZIP2, similar to AtZIP7, 8, and 11, MtZIP1, and MtZIP7 in *M. truncatula* (López-Millan et al., 2004), lacks this His-rich region between TM-III and TM-IV. Instead, it is found in their N-terminal, TM-IV, or TM-V as reported by Eng et al. (1998), indicating that these ZIPs may bind metal ions at a different site (Figure 1). PtZIP2, along with all the others, did contain the ZIP signature motif (consensus sequence: [LIVFA] [GAS] [LIVMD] [LIVSCG] [LIVFAS] [H] [SAN] [LIVFA] [LIVFMAT] [LIVDE] [G] [LIVF] [SAN] [LIVF] [GS]; Eng et al., 1998) in TM-IV (Figure 1). Taken together, all the identified characteristics of the PtZIPs suggest that they are reliable members of the ZIP family.

PtIRT1 shared 67.4% and 66.4% identity at the protein level with AtZIP10 and AtIRT1, respectively. Phylogenetic analysis also revealed that they were closely clustered together (Figure 2). PtIRT1 was also induced to a significantly greater extent in Fe-deficient roots (Figure 4) and complemented Zn, Fe, and Mn uptake-defective mutants (Figure 5), which was closely consistent with known functional characteristics of AtIRT1 (Korshunova et al., 1999; Rogers et al., 2000; Vert et al., 2002). This suggests that PtIRT1 is most likely the orthologous form of AtIRT1, so we officially name it “PtIRT1” herein. Phylogenetic analysis showed that although the closest orthologous could not be assigned, 6 PtZIPs (PtZIP1, PtZIP3, PtZIP5, PtZIP12, PtZIP13, and PtZIP14) resembled the cluster consisting of AtZIP1, AtZIP3, AtZIP5, and AtZIP12, which were mainly induced under Zn deficiency and found to be involved in Zn uptake and redistribution (Grotz et al., 1998; van de Mortel et al., 2006; Milner et al., 2013; Inaba et al., 2015). In the current results, four of them (PtZIP1, PtZIP3, PtZIP5, and PtZIP12) showed visible induction by Zn deficiency or complementation with *zrt1zrt2* (Figures 4, 5), indicating that these four PtZIPs may be functional orthologous of AtZIP1, AtZIP3, AtZIP5, and AtZIP12. Among them, PtZIP1 shared the highest identity (64.0%) with AtZIP1. They also showed a similar expression pattern under Zn deficiency and Zn uptake ability (Grotz et al., 1998). PtZIP5 was induced in Zn-deficient roots and leaves but it did not complement *zrt1zrt2*, which was similar to the known information of AtZIP5 (van de Mortel et al., 2006; Milner et al., 2013). PtZIP3 and PtZIP12, similar to AtZIP3 and AtZIP12 (Grotz et al., 1998; Milner et al., 2013), were able to complement *zrt1zrt2* but not *fet3fet4* or *smf1* (Figure 5), and PtZIP3 (57.8%) shared significantly closer identity with AtZIP3 than PtZIP12 did (49.7%). Based on this information, these four PtZIPs can be named PtZIP1, PtZIP3, PtZIP5, and PtZIP12, respectively. In this cluster, another two PtZIPs (PtZIP13 and PtZIP14) were neither assigned any close orthology nor found to have functional characteristics similar to those of known AtZIPs. We speculate that PtZIP13 and PtZIP14 may be previously unknown members of ZIP family in trifoliate orange relative to *A. thaliana*, and citrus plants may require more *ZIP* genes for Zn uptake or redistribution in organs, such as Zn loading in fruit. For this reason, they are here named PtZIP13 and PtZIP14, respectively. Based on phylogenetic analysis, AtZIP2, AtZIP7, AtZIP10, and AtZIP11 were clustered around only one closely related PtZIP, and the closely clustered two also shared the highest identity at protein level, for example 62.3% for PtZIP2 and AtZIP2, 67.7% for PtZIP6 and AtZIP6, 58.3% for PtZIP7 and AtZIP7, and 68.6% for PtZIP11 and AtZIP11. Thus, they are here named PtZIP2, PtZIP6, PtZIP7, and PtZIP11, respectively. For the PtZIP9, three closely AtZIPs (AtZIP4, AtZIP9, and AtIRT3) were clustered simultaneously (Figure 2). Previous studies have revealed that these three AtZIPs were visibly induced by Zn deficiency and two of them, AtZIP4 and AtIRT3, complemented *zrt1zrt2* mutant (van de Mortel et al., 2006; Lin et al., 2009; Assunção et al., 2010). In the current work, PtZIP9 was also been induced in Zn-deficient roots but failed to complement *zrt1zrt2*, which was consistent with existing reports on AtZIP9 (van de Mortel et al., 2006; Milner et al., 2013). Thus, it is called PtZIP9. It should be noted that although we have selected an appropriate name for 12 PtZIPs according to their sequence and functional comparability to *A. thaliana* ZIPs, our results and existing information concerning AtZIPs are still limited, so the relationship between PtZIPs and AtZIPs still needs to be identified through further study. As mentioned in results, a tandem duplication might have happened in the evolutionary history of PtZIP family since there are three pairs of *PtZIPs* neighboring on genome and also clustered together on phylogenetic tree. Tandem duplication of the genes in the genome evolution is commonly existing in organisms including citrus (Zhang, 2003; Xu et al., 2013). The duplicated genes are sometimes conserved in gene function, but more outcomes are the origin of novel function (Zhang, 2003). In this study, although three pairs of *PtZIPs* are both phylogenetically and physically close, no obvious similarity was found in their expression pattern and yeast complementing. Thus, we believe that these genes may have evolved their own functions.

Previous studies in *A. thaliana*, rice, soybeans, and maize have suggested the complicated expression profiles of *ZIP* genes in response to various metal ions and outlined their diverse functions in Zn, Fe, and Mn uptake, transport, and redistribution (Moreau et al., 2002; van de Mortel et al., 2006; Bashir et al., 2012, 2016; Milner et al., 2013; Li et al., 2015). PtZIPs also perform diverse functions in response to various metal ions. To establish these differences, it is necessary to comprehensively understand the results of the expression patterns and yeast complementation testing performed here. Results showed that *PtIRT1, PtZIP1, PtZIP2, PtZIP3, PtZIP5, PtZIP6*, and *PtZIP9* were induced mainly in Zn-deficient roots. Meanwhile, 4 of them (*PtIRT1, PtZIP1, PtZIP2*, and *PtZIP3*) complemented *zrt1zrt2* mutants visibly. This overlap suggests that at least these 4 PtZIPs may play a role in Zn uptake. Similarly, the corresponding *ZIPs* of *A. thaliana, AtIRT1, AtZIP1, AtZIP2*, and *AtZIP3*, were able to take up Zn (Grotz et al., 1998; Korshunova et al., 1999; Rogers et al., 2000). Under Fe deficiency, only *PtIRT1* and *PtZIP7* were induced mainly in roots. These two genes also strongly complemented *fet3fet4* mutants even under very strictly limited Fe availability. So we expect that *PtIRT1* and *PtZIP7* are responsible for high Fe uptake in trifoliate oranges. The corresponding *AtIRT1* has been well demonstrated in Fe uptake, but for *AtZIP7* only Milner et al. (2013) provided a few evidence which indicates its Fe uptake activity *via fet3fet4* complementation testing. The current findings regarding *PtZIP7* are completely consistent with previous studies. For the Mn uptake, although we did not find any overlapping results among expression pattern and yeast complementation, *PtIRT1* still showed prominent activity in complementing *smf1*. In *A. thaliana*, it has also been proved that *AtIRT1* could complement *smf1* and take up Mn (Korshunova et al., 1999; Rogers et al., 2000). We also noticed that the other four *PtZIPs* (*PtZIP1, PtZIP9, PtZIP11*, and *PtZIP13*) complemented growth of *smf1* in the initial 20 h (Figure 5D), but they failed to grow later. It seems that these genes only weakly complemented the growth of *smf1* mutant, but could not sustain proliferated yeast growth. Thus, out of the PtZIPs we identified, only PtIRT1 appears to be a Mn uptake transporter.

## Conclusion

In conclusion, 12 *PtZIP* genes were isolated from trifoliate orange plants, and sequence analysis and prediction suggests that they all possessed the basic characteristics of members of the ZIP family. Twelve *PtZIP* genes were identified and named *PtIRT1, PtZIP1, PtZIP2, PtZIP3, PtZIP5, PtZIP6, PtZIP7, PtZIP9, PtZIP11, PtZIP12, PtZIP13*, and *PtZIP14* based on the sequence and functional comparability to *A. thaliana* ZIPs. Comprehensively analyzing the results of expression patterns and yeast complementation tests indicates that *PtIRT1, PtZIP1, PtZIP2*, and *PtZIP3* are responsible for Zn uptake, *PtIRT1* and *PtZIP7* for Fe uptake, and *PtIRT1* for Mn uptake in trifoliate oranges. The present study provides essential information for citrus *ZIP* genes, but further work is needed to characterize the exact subcellular and tissue localization, transcriptional regulation, and functions of the *PtZIP* genes in the different citrus species.

## Author contributions

XF and LP conceived and designed the study. XF, XZ, FX, LL, CC, and LC performed the experiments. XF, XZ, and MA analyzed the data. XF wrote the manuscript and MA revised it. All authors have read and approved the final manuscript.

### Conflict of interest statement

The authors declare that the research was conducted in the absence of any commercial or financial relationships that could be construed as a potential conflict of interest.
